# The metabolic outcomes of growth hormone treatment in children are gender specific

**DOI:** 10.1530/EC-18-0135

**Published:** 2018-06-20

**Authors:** Alessandro Ciresi, Stefano Radellini, Valentina Guarnotta, Maria Grazia Mineo, Carla Giordano

**Affiliations:** Section of EndocrinologyBiomedical Department of Internal and Specialist Medicine (DIBIMIS), University of Palermo, Palermo, Italy

**Keywords:** growth hormone, gender, children, insulin sensitivity, metabolism

## Abstract

**Objective:**

To evaluate the impact of gender on the clinical and metabolic parameters in prepubertal growth hormone deficiency (GHD) children at diagnosis and during GH treatment (GHT).

**Design:**

The data of 105 prepubertal children (61 males, 44 females, mean age 6.8 ± 0.7 years) affected by idiopathic GHD were retrospectively evaluated.

**Methods:**

Body height, BMI, waist circumference (WC), IGF-I, HbA1c, lipid profile, fasting and after-OGTT glucose and insulin levels, insulin sensitivity and secretion indices were evaluated at baseline and after 24 months of GHT.

**Results:**

At baseline, no significant difference was found in all clinical, hormonal and metabolic parameters between males and females. After 24 months of GHT, both males and females showed a significant increase in height (both *P* < 0.001), BMI (both *P* < 0.001), WC (*P* < 0.001 and *P* = 0.004, respectively), IGF-I (both *P* < 0.001), fasting glucose (*P* < 0.001 and *P* = 0.001, respectively), fasting insulin (both *P* < 0.001) and Homa-IR (both *P* < 0.001), with a concomitant significant decrease in insulin sensitivity index (ISI) (both *P* < 0.001) and oral disposition index (DIo) (*P* = 0.001 and *P* < 0.001, respectively). At 24 months of GHT, females showed significantly higher BMI (*P* = 0.027), lower ISI (*P* < 0.001) and DIo (*P* < 0.001), in concomitance with a significant greater change from baseline to 24 months of BMI (*P* = 0.013), WC (*P* < 0.001), ISI (*P* = 0.002) and DIo (*P* = 0.072), although the latter does not reach statistical significance.

**Conclusions:**

Twenty-four months of GHT in prepubertal children leads to different metabolic outcomes according to gender, with a greater reduction in insulin sensitivity in females, regardless of auxological and hormonal parameters. Therefore, prepubertal GHD females should probably need a more proper monitoring in clinical practice.

## Introduction

The sexual dimorphism in the mechanisms involved in the regulation of somatotroph axis is well documented, and it might account for some of the sex differences in growth rate and body composition (1). Indeed, sex hormones are one of the most influential regulators of growth hormone (GH) secretion and action, with different mechanisms (2). Androgens and estrogens exert opposite endocrine-mediated effects on insulin-like growth factor (IGF)-I production and metabolic parameters. Androgens affect circulating IGF-I indirectly via increased GH secretion, thus enhancing the peripheral GH action. In addition, testosterone modulates peripheral action of GH on the growth plate and liver by enhancing GH receptor expression (3). Conversely, estrogens reduce hepatic IGF-I production, consequently enhancing GH secretion and inhibit the function of the GH receptor (4). In addition, somatostatin also plays a role in the sexual dimorphism of GH secretion (5). Indeed, high levels of estrogen receptors are expressed in the pituitary, regulating GH gene expression and reducing somatostatin receptor expression, leading to enhanced GH secretion (6). Lastly, the role of gender and sex steroids in modulating the clearance of GH must be taken into account (7). Therefore, women have higher GH secretion rates, due to both higher amplitude and higher frequency of the secretory pattern, and both baseline and post-glucose GH nadir concentrations are greater in women than in men (8, 9).

In healthy subjects, GH and IGF-I levels decline significantly with age in both sexes, while men show lower baseline and mean GH levels than women mainly due to the action of estradiol on the neuroendocrine regulation of pulsatile GH release (10).

This complex interplay between sex hormones and the GH/IGF-I axis has a clinically relevant impact in patients with hypopituitarism and the gender-dimorphic GH secretion pattern must be taken into account both in diagnosis of GH deficiency (GHD) and in detection of the appropriate and effective dose of GH replacement treatment (GHT) to be used (11).

Indeed, in hypopituitary patients, GHT produces a greater increase in plasma IGF-I in men, and women require a higher GH dose due to lower responsiveness to standard GH doses (12, 13).

The sexual dimorphism of GH secretion also results in a different expression of some genes involved in glucose and lipid metabolism. There is strong evidence that estrogens can influence the GH-regulated endocrine and metabolic functions in the human liver, and testosterone maximizes the metabolic benefits of GH (2, 14).

Height and weight gain or body composition become sexually dimorphic around the time of puberty, when males more rapidly gain muscle and bone and lose fat than females (1) and the close relationship between GH and gonadal steroid levels during puberty suggests that gonadal steroids may also stimulate linear growth by enhancing pituitary GH secretion (15).

Conversely, the relationship between GH and gonadal steroid levels in children is difficult to establish because steroid levels are very low prior to puberty. Therefore, although the clinical features related to sexual dimorphism of the somatotroph axis have been well demonstrated in GHD adults, to date, very few studies about gender difference are available in prepubertal GHD children.

The aim of this study was to evaluate the impact of gender on the clinical and metabolic parameters in a large series of prepubertal GHD children at diagnosis and during GHT.

## Subjects and methods

We retrospectively evaluated the data of 105 children (61 males, 44 females, mean age 6.8 ± 0.7 years; range 4.3–9.4) affected by idiopathic GHD who were consecutively admitted to the Section of Endocrinology of the University of Palermo from January 2013 to December 2017 and underwent GHT for at least 24 consecutive months. Fifty-one healthy subjects, matched for sex (33 males, 18 females), age (mean age 6.7 ± 0.6 years; range 4–7.8), stature and pubertal status, were recruited as a control group at baseline among children referred for short stature. In this group, screening for short stature did not reveal endocrine disease and GHD was excluded. We excluded children affected by organic GHD or multiple pituitary hormone deficiency or receiving other hormonal replacement treatment to avoid interference with the metabolic parameters evaluated. All children, including the older ones, were in the first stage of sexual development according to the criteria of Marshall and Tanner (girls with Tanner breast stage B1; boys with testicular volume <4 mL) to avoid any interference of puberty with the auxological and metabolic parameters analyzed, and they maintained their prepubertal clinical and hormonal status for the entire follow-up (i.e. FSH and LH <1 U/mL, total testosterone and 17β-estradiol <0.50 ng/mL and <5 pg/mL in males and females, respectively). Similarly, no signs of clinical or biochemical adrenarche were detected during the entire follow-up (i.e. dehydroepiandrosterone sulphate <0.48 μmol/L or 18 µg/dL in all children). GHD was diagnosed according to the criteria of the GH Research Society (16).

Specifically, as auxological criteria, we considered height more than 2 standard deviations (s.d.) below the mean and growth velocity more than 1 s.d. below the mean for age, or, without severe short stature, a growth velocity more than 2 s.d. below the mean over 1 year. As radiological criteria, we considered a bone age delay, estimated from an X-ray of the left wrist and hand and evaluated according to the methods of Greulich and Pyle, of at least 1 year with respect to the chronological age (17).

Biochemically, GHD was diagnosed by failure of GH to respond to the glucagon stimulation test (GST) and the insulin tolerance test (ITT), with GH peaks below 8 µg/L according to the Italian criteria of appropriateness of use and reimbursement of GHT in children (18).

Neuroimaging, with magnetic resonance of the hypothalamic–pituitary region, was performed in all GHD children with more severe GHD (GH peak ≤3 μg/L) and did not show significant pituitary abnormalities.

All GHD children received GH once daily at bedtime with a pen injection system. IGF-I levels and growth velocity allowed us to determine the GH dose. On average, in all patients, the initial daily dose of GH was 0.025 mg/kg, and it was gradually increased to 0.027–0.028 mg/kg from month 6 to 12, to 0.029–0.030 mg/kg from month 12 to 18 and to 0.030–0.032 mg/kg from month 18 to 24 in order to achieve the above-mentioned targets.

### Study protocol

In all children at baseline, we measured body height (expressed as s.d.), BMI s.d. and waist circumference (WC). GH secretion was evaluated by GH levels during GST and ITT, performed on two different days. During GST, blood samples were collected at 0, 30, 60, 90, 120, 150, 180 and 240 min after the injection of 30 μg/kg (up to 1000 μg) intramuscularly of glucagon (GlucaGen, NovoNordisk), for measurements of GH. Blood samples for GH were also measured during ITT (0.1 U/kg of body weight of human Humulin R insulin) at baseline and at 30, 60, 90 and 120 min after insulin administration and results were accepted with blood glucose lower than 2.2 nmol/L (40 mg/dL).

On a different day, a blood sample was drawn after an overnight fast for the measurement of hemoglobin A1c (HbA1c), IGF-I concentrations and lipid profile, including total and high-density lipoprotein (HDL) cholesterol and triglycerides. Low-density lipoprotein (LDL) cholesterol levels were evaluated by the following formula: total cholesterol–(HDL cholesterol–triglycerides/5). This sample also served as the baseline sample for an oral glucose tolerance test (OGTT). Blood samples were collected every 30 min for 2 h for glucose and insulin measurements.

As surrogate estimates of insulin sensitivity we considered the homeostasis model assessment estimate of insulin resistance (Homa-IR) (19) and the insulin sensitivity index (ISI), a composite index derived from the OGTT and validated by Matsuda and DeFronzo (20). The oral disposition index (DIo) was used to evaluate the ability of the β-cell to regulate its insulin response to stimuli based on differences in insulin sensitivity. The DIo was calculated at the time 0′ and 30′ during OGTT as described (21), using the following formula, where insulin levels are expressed in IU/mL and glucose levels in mmol/L: DIo = (Δ insulin 0′–30′/Δ glucose 0′–30′) × 1/fasting insulin.

After the diagnosis of GHD was made, in GHD children in addition to auxological parameters and IGF-I measurement we performed OGTT (for glucose and insulin) after 12 and 24 months of GHT. In the control group, these evaluations were only performed at baseline.

For the analyses, we also considered the change (delta) in clinical and metabolic parameters from baseline to 24 months of GHT. The Institutional Ethics Committee of the University of Palermo approved this study. At the time of hospitalization, an informed consent for the scientific use of the data was obtained from both the participants and their parents.

### Hormone and biochemical assays

Glucose was measured in the centralized accredited laboratories of the University of Palermo with the standard methods. HbA1c levels were determined by HPLC with an ion-exchange resin (BioRad D10, BioRad). Serum insulin was measured by electrochemiluminescence (ECLIA, Elecsys Insulin, Roche). The sensitivity of the method was 0.4 µU/mL. The normal range (µU/mL) was 2.6–24.9. Serum GH levels were measured by Immunoassay in electrochemiluminescence (ECLIA, Elecsys hGH, Roche). The lower limit of detection of the assay was 0.030 µg/L. The intra- and inter-assay coefficients of variation (CV) were 0.6–5.0 and 3.8–5.0%, respectively. We reported GH concentrations in µg/L of IS 98/574. Serum IGF-I levels were measured by means of a chemiluminescent immunometric assay (Immulite 2000; Diagnostic Products Corp., Los Angeles, CA, USA) using murine monoclonal anti-IGF-I antibodies. The standards were calibrated against the World Health Organization second IS 87/518. The sensitivity was 1.9 µg/L. The intra- and inter-assay CVs were 2.3–3.9% and 3.7–8.1%, respectively.

### Statistical analysis

The Statistical Packages for Social Sciences SPSS, version 19 was used for data analysis. Baseline characteristics were presented as mean ± s.d. for continuous variables (normality of distribution for the quantitative variables was assessed with the Kolmogorov–Smirnov test); rates and proportions were calculated for categorical data. The differences between groups were evaluated with the *t*-test. A *P* value <0.05 was considered statistically significant.

## Results

### Children with GHD vs controls

The clinical, hormonal and metabolic parameters of control subjects and GHD children in full at diagnosis and after 24 months of GHT are shown in Table 1.

**Table 1:** Clinical, hormonal and metabolic parameters of control subjects and GHD children in full at diagnosis and after 24 months of GH treatment.

	Controls *No 51*	GHD (baseline) *No 105*	GHD (24 months) *No 105*	*P*	P*
	Subjects (%)	Subjects (%)			
Gender				0.130	–
Males	33 (65)	61 (58)			
Females	18 (35)	44 (42)			
	Mean ± s.d.	Mean ± s.d.			
Age (years)	6.7 ± 0.6	6.8 ± 0.7	–	0.424	–
Height (s.d.)	−1.9 ± 0.3	−2.3 ± 0.4	−1.4 ± 0.4	<0.001	<0.001
BMI (s.d.)	−0.5 ± 0.2	−0.7 ± 0.5	−0.4 ± 0.4	0.130	<0.001
Waist circumference (cm)	59 ± 7	62 ± 10	65 ± 9	0.101	<0.001
GH peak during GST (µg/L)	11.2 ± 6.1	3.5 ± 2.3	–	<0.001	–
GH peak during ITT (µg/L)	14.9 ± 7.6	4.5 ± 2.5	–	<0.001	–
IGF-I (s.d.)	0.3 ± 0.4	−0.3 ± 0.5	0.4 ± 0.5	<0.001	<0.001
Fasting glucose (mmol/L)	4.1 ± 0.3	4.1 ± 0.6	4.5 ± 0.4	0.871	<0.001
Fasting insulin (µU/mL)	3.7 ± 3.1	3.8 ± 3.3	8.6 ± 4.6	0.906	<0.001
HbA1c (%)	5.2 ± 0.2	5.1 ± 0.2	5.1 ± 0.2	0.372	0.990
Homa-IR	0.7 ± 0.6	0.7 ± 0.7	1.7 ± 1	0.679	<0.001
ISI	10.1 ± 3.3	11.1 ± 4	6.9 ± 4.2	0.125	<0.001
DIo	6 ± 3.6	5 ± 3.5	2.9 ± 2	0.111	<0.001
Total cholesterol (mmol/L)	4.1 ± 0.6	4.1 ± 0.7	4.1 ± 0.6	0.981	0.480
HDL cholesterol (mmol/L)	1.7 ± 0.4	1.6 ± 0.3	1.6 ± 0.3	0.149	0.569
LDL cholesterol (mmol/L)	2 ± 0.6	2.2 ± 0.6	2.1 ± 0.5	0.285	0.144
Triglycerides (mmol/L)	1.6 ± 0.7	1.5 ± 0.5	1.6 ± 0.5	0.284	0.108

*P* = difference between controls and GHD children at baseline; P* = difference between GHD children at baseline and after 24 months of GH treatment.

BMI, body mass index; DIo, oral disposition index; GST, glucagon stimulation test; Homa-IR, homeostasis model assessment estimate of insulin resistance; ISI, insulin sensitivity index; ITT, insulin tolerance test; s.d., standard deviation; WC, waist circumference.

At baseline, GHD children showed significantly lower height (*P* < 0.001), IGF-I (*P* < 0.001) and GH peak after GST (*P* < 0.001) and ITT (*P* < 0.001) than control subjects. No significant difference in BMI and WC between GHD children and controls was found. Similarly, no difference was found in all metabolic parameters analyzed (Table 1).

After 24 months of GHT, GHD children showed a significant increase in height (*P* < 0.001), BMI (*P* < 0.001), WC (*P* < 0.001) and IGF-I (*P* < 0.001).

Regarding the metabolic parameters, GHD children showed a significant increase in fasting glucose (*P* < 0.001), fasting insulin (*P* < 0.001) and Homa-IR (*P* < 0.001), with a concomitant significant decrease in ISI (*P* < 0.001) and DIo (*P* < 0.001). No significant change was found in Hba1c levels and lipid profile (Table 1).

### GHD children grouped according to gender

The gender-specific clinical, hormonal and metabolic parameters of GHD children at diagnosis and after 24 months of GHT are shown in Table 2.

**Table 2:** Gender-specific clinical, hormonal and metabolic parameters of GHD children at diagnosis and after 24 months of GH treatment.

	Males		*P*	Females		P^*^	P**	P***
	Baseline	24 months		Baseline	24 months			
	Mean ± s.d.	Mean ± s.d.		Mean ± s.d.	Mean ± s.d.			
Age (years)	6.7 ± 0.5	–	–	6.9 ± 0.9	–	–	0.233	–
Height (s.d.)	−2.2 ± 0.3	−1.4 ± 0.3	<0.001	−2.3 ± 0.4	−1.4 ± 0.4	<0.001	0.168	0.608
BMI (s.d.)	−0.8 ± 0.5	−0.6 ± 0.4	<0.001	−0.6 ± 0.4	−0.2 ± 0.3	<0.001	0.246	0.027
WC (cm)	62 ± 10	64 ± 8.5	0.004	62 ± 10	67 ± 9.4	<0.001	0.967	0.059
GST-GH peak (µg/L)	3.8 ± 2.2	–	–	2.9 ± 2.4	–	–	0.067	–
ITT-GH peak (µg/L)	4.2 ± 2.6	–	–	4.9 ± 2.3	–	–	0.170	–
IGF-I (s.d.)	−0.3 ± 0.4	0.4 ± 0.4	<0.001	−0.3 ± 0.5	0.4 ± 0.5	<0.001	0.495	0.623
Fasting glucose (mmol/L)	4.2 ± 0.4	4.5 ± 0.4	<0.001	4 ± 0.7	4.5 ± 0.4	0.001	0.093	0.587
Fasting insulin (µU/mL)	3.3 ± 3.1	8.4 ± 4.4	<0.001	4.3 ± 3.5	9 ± 4.9	<0.001	0.137	0.514
HbA1c (%)	5.2 ± 0.2	5.2 ± 0.2	0.744	5.1 ± 0.2	5.1 ± 0.3	0.739	0.158	0.295
Homa-IR	0.6 ± 0.6	1.7 ± 1	<0.001	0.8 ± 0.7	1.8 ± 1	<0.001	0.178	0.657
ISI	11.5 ± 3.8	8 ± 4.3	<0.001	10.6 ± 4.2	5.9 ± 4.2	<0.001	0.286	<0.001
DIo	5.3 ± 3.8	3.5 ± 2.1	0.001	4.7 ± 3	2.1 ± 1.5	<0.001	0.424	<0.001
Total cholesterol (mmol/L)	4.1 ± 0.8	4.1 ± 0.7	0.137	4.1 ± 0.6	4.2 ± 0.6	0.248	0.838	0.458
HDL cholesterol (mmol/L)	1.6 ± 0.3	1.6 ± 0.3	0.781	1.5 ± 0.3	1.6 ± 0.4	0.260	0.105	0.754
LDL cholesterol (mmol/L)	2.1 ± 0.7	2.1 ± 0.4	0.179	2.2 ± 0.5	2.1 ± 0.6	0.585	0.672	0.566
Triglycerides (mmol/L)	1.4 ± 0.5	1.6 ± 0.4	0.217	1.5 ± 0.4	1.6 ± 0.5	0.307	0.218	0.647

*P* = difference from baseline to 24 months of GH treatment in males; P* = difference from baseline to 24 months of GH treatment in females; P** = difference between males and females at baseline; P** = difference between males and females at 24 months of GH treatment.

BMI, body mass index; DIo, oral disposition index; GST, glucagon stimulation test; Homa-IR, homeostasis model assessment estimate of insulin resistance; ISI, insulin sensitivity index; ITT, insulin tolerance test; s.d., standard deviation; WC, waist circumference.

At baseline, no significant difference was found in all clinical, hormonal and metabolic parameters between males and females (Table 2).

After 24 months of GHT, both males and females showed a significant increase in height (both *P* < 0.001), BMI (both *P* < 0.001), WC (*P* < 0.001 and *P* = 0.004, respectively) and IGF-I (both *P* < 0.001).

Regarding the metabolic parameters, in both males and females, we observed a significant increase in fasting glucose (*P* < 0.001 and *P* = 0.001, respectively), fasting insulin (both *P* < 0.001) and Homa-IR (both *P* < 0.001), with a concomitant significant decrease in both sexes in ISI (both *P* < 0.001) and DIo (*P* = 0.001 and *P* < 0.001, respectively). No significant change was found in Hba1c levels and lipid profile in either males or females (Table 2).

When we directly compared the clinical, hormonal and metabolic parameters at 24 months in GHD children according to gender, females showed significantly higher BMI (*P* = 0.027), lower ISI (*P* < 0.001) and DIo (*P* < 0.001) and higher values of WC (*P* = 0.059), although not statistically significant (Table 2), while no significant difference was found in height, IGF-I, fasting glucose, fasting insulin, HbA1c, Homa-IR and lipids profile.

**Figure 1:**
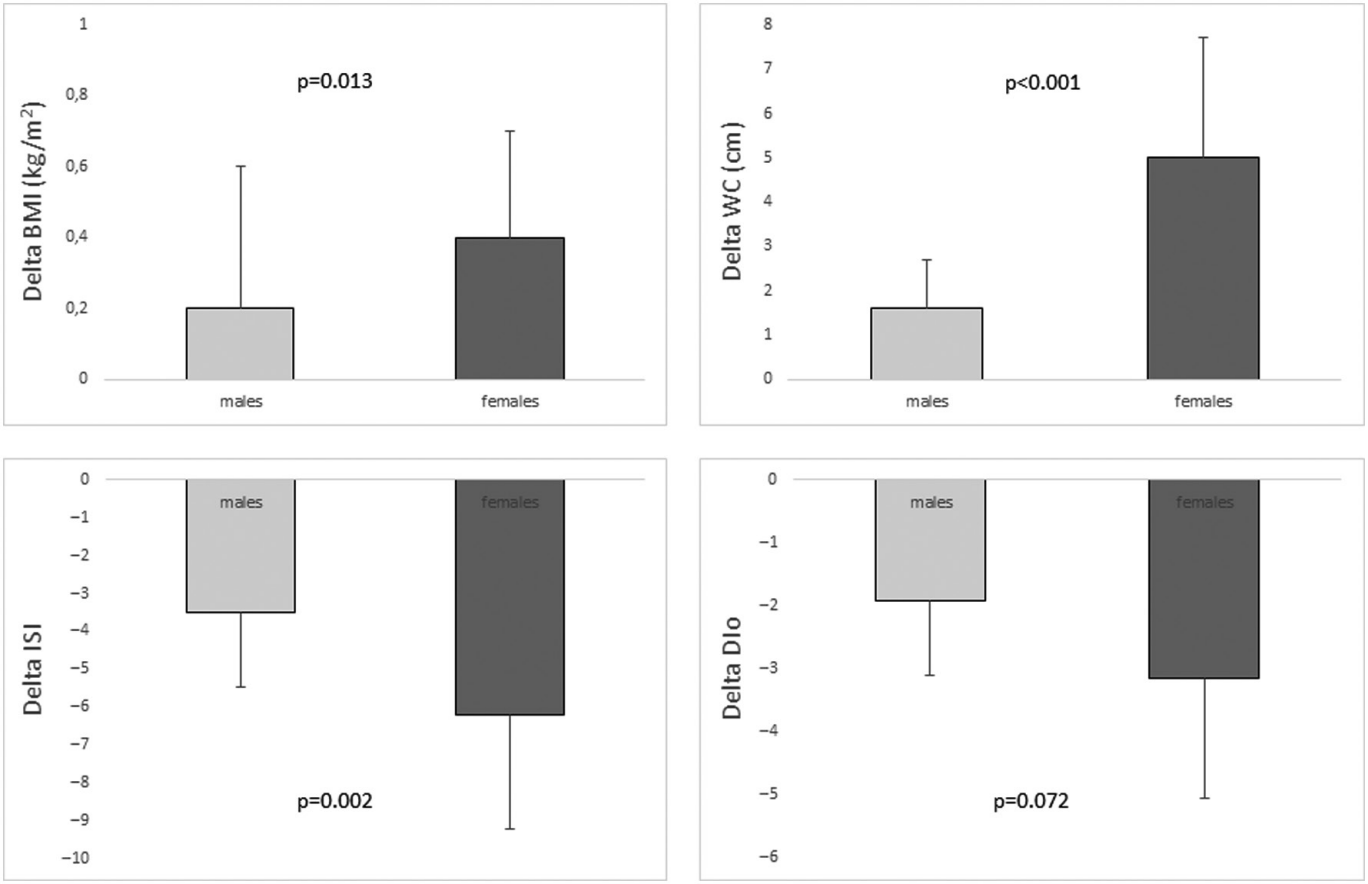
Change (delta) in BMI, waist circumference (WC), insulin sensitivity index (ISI) and oral disposition index (DIo) from baseline to 24 months of GH treatment in GHD children grouped according to gender.

In addition, the comparison of delta of all parameters from baseline to 24 months showed significantly greater delta of BMI (0.4 ± 0.3 vs 0.2 ± 0.4 s.d.; *P* = 0.013), WC (5 ± 2.7 vs 1.6 ± 4.1 cm; *P* < 0.001) and ISI (−6.2 ± 5 vs −3.5 ± 4.6; *P* = 0.002) and greater delta of DIo (−3.1 ± 2.9 vs −1.9 ± 3.9; *P* = 0.072) in females, although the latter does not reach statistical significance (Fig. 1), without other significant differences (data without significant difference are not shown).

## Discussion

In this study, we found that prepubertal GHD females show a worse metabolic profile than males after 24 months of GHT, regardless of the auxological and hormonal data, indicating that gender may impact on the metabolic outcomes during GHT also in children. Indeed, although all children showed a significant improvement in auxological data and IGF-I levels in concomitance with a slight deterioration in glucose metabolism, insulin sensitivity and β-cell response after GHT, females demonstrated a greater reduction in insulin sensitivity and pancreatic function, probably related to higher increase in fat mass.

The sexual dimorphism of the somatroph axis has been well documented in healthy subjects and in adult patients affected by both GH excess and deficiency (12, 22, 23, 24). GHD adolescents also showed a gender difference in the clinical effect of treatment, since in the transition phase, GHT determined a greater gain in lean body mass and reduction in fat mass in males than in females (25), although not all studies are concordant. In fact, Mauras *et al.* showed similar sensitivity to GHT for body composition changes, lipolysis, lipid and glucose concentrations in males and females during puberty and just a higher IGF-I response in males, which does not translate into differences in linear growth (26). The effect of gender on clinical response to GHT in children has been poorly explored. Although in the present study all children were prepubertal during the entire follow-up, as demonstrated by both clinical and hormonal parameters, previous studies demonstrated that sex steroid levels in children, although much lower, may display sexual dimorphism. Indeed, prepubertal girls show higher estrogen levels than prepubertal boys, and moreover, boys have higher testosterone levels compared to prepubertal girls (27). Therefore, very low levels of sex steroids can probably also modulate the GH axis or clinical response to GHT.

Rose *et al.* in a retrospective study evaluated whether this was a sex steroid effect during the first 2 years of GHT in 147 prepubertal children, finding no evidence of gender difference in growth response (28).

Similarly, no significant gender differences in metabolic parameters were observed both at baseline and during GHT by Salerno *et al.* (29). Conversely, Cohen *et al.* in a randomized prospective dose-ranging study demonstrated a clear gender impact on both auxological and biochemical parameters in a larger cohort of GHD children during GHT, concluding that there is a gender difference in GH sensitivity. Indeed, the authors showed that males had a linear GH dose response, whereas females had an apparent plateau of both growth and IGF-I levels, and these data were also confirmed when only prebubertal children were analyzed, indicating that the gender difference observed is not related to the occurrence of puberty (30). Savendahl *et al*. also reported a significant gender difference in 2-year growth response to GHT in a large cohort of prepubertal GHD children, although no difference was observed in the change in IGF-I levels (31).

In our study, both GHD children taken together and grouped according to gender showed a significant and similar auxological improvement and normalization of IGF-I levels during GHT. However, if apparently all children had increased BMI and WC, the direct comparison between males and females after 24 months of GHT showed a greater increase in fat mass in females, as confirmed by the calculated delta of BMI and WC. These data are in agreement with a previous study by Kuromaru *et al.* who demonstrated similar auxological benefits but partial gender-specific metabolic effects of GHT in 62 children. Indeed, the authors showed a significant change in percent body fat both in boys and girls during the first 6 months of treatment, although body fat remained constant in boys but started to increase in girls from the 18th month of treatment (32).

Few studies have evaluated the gender differences of metabolic features in children with GHD. GHT is known to impact on glucose metabolism, by increasing insulin secretion and decreasing insulin sensitivity, with a discordant impact on glucose levels (33, 34). In the present study, GHD children at baseline did not show any significant metabolic difference from controls, in agreement with previous studies (35, 36, 37).

After 24 months of GHT, regardless of gender, all GHD children showed a significant increase in fasting glucose and insulin levels with a concomitant impairment in insulin sensitivity and β-cell function. Indeed, although fasting glucose and HbA1c levels were always maintained within the normal range, a significant decrease in insulin sensitivity, demonstrated by an increase in Homa-IR and a decrease in ISI, was found in all children after GHT. These data are quite consistent with the majority of existing studies (38, 39, 40, 41, 42, 43). In addition, a significant decrease in the DIo was found in all children, in agreement with a previous study (44). The DIo, which expresses the capacity of β-cells to compensate adequately for insulin resistance through increased insulin secretion, has been shown to be a predictor of development of diabetes (21). Therefore, its reduction is considered an index of inadequate β-cell capacity to counteract the reduction in insulin sensitivity and a risk factor for future development of overt diabetes (45). However, when we considered the change in metabolic parameters according to gender, females showed a greater deterioration in insulin sensitivity, as demonstrated by the higher reduction in ISI, with a concomitant greater reduction in DIo, while no gender difference was found in IGF-I levels.

Therefore, the gender difference in the metabolic outcomes of GHT observed in our patients were unlikely due to being mediated by the IGF-I levels, which were always within the normal range during the observational period and similar in males and females. However, to minimize the impact of the different age of patients on IGF-I, we considered the delta in IGF-I during GHT, rather than absolute IGF-I levels, and it too proved comparable in the two groups of children. Similarly, a different GH dose in males and females can be excluded as the cause of the different metabolic outcomes in this study, since the dose was similar in both groups of patients during the entire follow-up. This finding suggests that there are other factors than IGF-I change or different GH dose that may mediate the gender difference in metabolic outcomes during GHT.

Notably, in our study, females showed a greater increase in BMI and WC than males, as demonstrated by the comparison of these parameters at 24 months and as confirmed by the significant difference in delta of BMI and WC between the two groups. Therefore, the greater change in BMI and WC found in females suggests that adiposity might be a mediator of the different metabolic effects of GHT.

Indeed, a different gender response to GHT secondary to body composition changes has been widely demonstrated in GHD adults (46, 47), although not all studies are concordant with a gender-related difference in the long-term metabolic effects of GHT (48). Conversely, the potential impact of adrenal androgen synthesis, which has also been demonstrated to be linked to GH/IGF-I axis and metabolic parameters in prepuberty (27), may be ruled out in our study.

This study has undoubtedly some limitations represented by the relative small size of the population and by the lack of data for the control group during the 24 months of follow-up. Indeed, the insulin sensitivity seems to decrease with age, probably due to the physiological effect of steroids production or the modifications of body composition during the peripubertal period, also in normal children (49, 50). For these reasons, it is not possible to determine with absolute certainty whether the greater increase in BMI and WC and decrease in ISI shown in females are exclusively related to GHT or would have occurred anyway, although in our study, all children remained clinically and biochemically prepubertal during the entire follow-up. More reliable data on body composition evaluated by dual energy X-ray absorptiometry (DEXA) will certainly confirm these important data in future studies. In addition, a close link between the decrease of DIo and the risk for diabetes in this population of GHD children can only demonstrated through longitudinal studies, and it would be interesting to know what happens to DIo after GHT will be discontinued. Conversely, the strength of this study lies in the fact that it evaluated the gender difference in metabolic outcomes of GHT in children as its primary and main objective.

In conclusion, this study highlights that 24 months of GHT in children leads to different metabolic outcomes according to gender, regardless of auxological and hormonal parameters. Therefore, the follow-up of prepubertal GHD children during GHT should probably also be modulated and optimized not only according to auxological and biochemical response, but also considering the patient’s gender. Specifically, prepubertal GHD females should probably need a more proper monitoring in clinical practice aimed at correct lifestyle during GHT to prevent metabolic alterations, although studies with longer follow-up and a larger cohort of patients are required to confirm these preliminary results and to establish with greater certainty whether GHD females need more surveillance during GHT or, alternatively, different GHT doses.

## Declaration of interest

The authors declare that there is no conflict of interest that could be perceived as prejudicing the impartiality of the research reported.

## Funding

This research did not receive any specific grant from any funding agency in the public, commercial or not-for-profit sector.
